# Accumulation of ^111^In-Labelled EGF-Au-PEG Nanoparticles in EGFR-Positive Tumours is Enhanced by Coadministration of Targeting Ligand

**DOI:** 10.7150/ntno.19952

**Published:** 2017-06-08

**Authors:** Lei Song, Sarah Able, Errin Johnson, Katherine A. Vallis

**Affiliations:** 1 CR-UK/MRC Oxford Institute for Radiation Oncology, Department of Oncology, University of Oxford, Oxford, OX3 7DQ, UK;; 2 Sir William Dunn School of Pathology, University of Oxford, Oxford, OX1 3RE, UK.

**Keywords:** gold nanoparticle, EGF, radiolabelling, ^111^In, cancer targeting, coadministration.

## Abstract

The successful use of targeted radionuclide therapy in the treatment of solid tumours may be limited by radioresistance, which necessitates delivery of a high dose of radioactivity. Nanoparticle (NP)-based delivery systems possess a large surface area for attachment of radioisotopes and so offer a solution to this challenge. However, tumour uptake may be limited by rapid hepatic clearance of NP via the mononuclear phagocyte system. Liver uptake is further compounded when epidermal growth factor (EGF) is used as a targeting ligand, as EGF-tagged NP bind the EGF receptor (EGFR), which is expressed to a moderate extent by hepatocytes. This report describes an indium-111 (^111^In)-labelled PEGylated EGF-tagged gold (Au) NP (^111^In-EGF-Au-PEG) and an effective strategy of coadministration of targeting ligand to address these issues. Direct attachment of EGF to the surface of Au NP did not compromise surface coating with long-chain PEG. *In vitro* experiments showed that ^111^In-EGF-Au-PEG targets EGFR-positive cancer cells (MDA-MB-468): >11% of radioactivity was internalised after incubation for 4 h. In *in vivo* studies accumulation of NP was observed in MDA-MB-468 xenografts and tumour uptake was enhanced by the coadministration of 15 µg of the unlabelled targeting ligand, EGF, to block hepatic EGFR. Uptake was 3.9% versus 2.8% injected dose/g (%ID/g) of tumour tissue with and without unlabelled EGF, respectively. Coadministration of EGF reduced liver uptake by 25.95% to 7.56 %ID/g. This suggests that the coadministration of unlabelled targeting ligand with radiolabelled PEGylated NP offers a promising strategy for targeting EGFR-positive cancer and for minimising liver uptake.

## Introduction

Targeted radionuclide therapy (TRT), such as radioimmunotherapy, which uses radiolabelled antibodies as targeted therapeutics, has been used effectively in the treatment of haematological malignancies, such as leukaemia and lymphoma. The aim of TRT is to deliver a cytotoxic dose of radioactivity to cancer cells with minimal radiation exposure to normal tissue. For example, Zevalin® (^90^Y-ibritumomab tiuxetan) and Bexxar® (^131^I-tositumomab), which target the CD20 antigen expressed by B-cells, have shown promising results in non-Hodgkin lymphoma 1-3. However, TRT is often less effective in the treatment of solid tumours, which are more radioresistant than leukaemia and lymphoma. Indeed, it has been suggested that the radiation doses needed to eliminate solid tumours are about 5-10 times greater than those necessary for the effective treatment of lymphoma and leukaemia 4. Therefore, following systemic administration, the amount of radioactivity delivered to the tumour by a radiolabelled monoclonal antibody or other targeting ligand may be insufficient to effectively eradicate solid tumours. It is possible to increase the amount of radioactivity that specifically reaches the tumour by direct intratumoural injection 5, 6. However, such an approach is most applicable to superficial tumours, which account for a minority of solid tumours. Hence, TRT of solid tumours remains a technical and dosimetric challenge.

In recent years nanotechnological approaches have been applied to the delivery of TRT 6-9. The use of nanoparticles (NP), in the size range 1 to 100 nm, as drug delivery systems has been the subject of intense research, particularly for cancer diagnosis and treatment 10, 11. NP selectively accumulate in cancers through the enhanced permeability and retention effect (EPR), a unique characteristic of tumours. The first FDA-approved nanomedicine was Doxil® (doxorubicin-loaded liposomes), which has been in clinical use since the 1990s 12. NP can carry a large payload given their high surface area to volume ratio. In addition, the extensive surface area offers an opportunity to attach other molecules such as surface coating materials, targeting ligands and drugs, which can influence the *in vivo* pharmacokinetics, targeting and therapeutic characteristics of the NP 13, 14. Furthermore, NP themselves, particularly inorganic NP, have unique optical, electronic, magnetic and biological properties, that can be exploited to enable their use in imaging or theranostic applications 15, 16.

We have previously synthesised epidermal growth factor (EGF)-tagged gold (Au) NP (i.e. ^111^In-EGF-Au) which were designed for the molecular radiotherapy of EGF receptor (EGFR)-positive cancer 17. Au NP were chosen as the basis for the development of the ^111^In-labelled targeted nanosystem because of their controllable, uniform size and ease of functionalisation 18-21. These advantages have led to Au NP-based products progressing into clinical trials 22, 23. We previously reported that ^111^In-EGF-Au NP delivered a significant amount of radioactivity to EGFR-positive cancer cells *in vitro*, leading to radiotoxicity 17. In this report, the *in vivo* biodistribution of ^111^In-EGF-Au NP was investigated using two mouse xenograft models, MDA-MB-468 (1.3 x 10^6^ EGFR/cell) and MDA-MB-231-H2N (hereafter referred to as 231-H2N) (2 x 10^5^ EGFR/cell).

One challenge in developing NP delivery systems is the rapid clearance of NP from the circulation via the mononuclear phagocyte system (MPS). Blood plasma proteins become adsorbed on to the surface of NP, a process termed opsonisation, which renders the particles visible to phagocytic cells in the liver and spleen. The surface coating of NP can significantly influence the opsonisation process 24, 25. PEGylation, for example, has been shown to inhibit opsonisation 26, 27.

Here we report the synthesis of PEGylated ^111^In-EGF-Au (^111^In-EGF-Au-PEG) through direct sequential attachment of EGF and PEG onto the surface of Au NP. Due to its relatively small size, directly tagged EGF is not expected to compromise surface coating with long-chain PEG. PEGylation, in turn, is expected to result in improved pharmacokinetics and biodistribution of ^111^In-EGF-Au-PEG. The targeting properties of PEG-coated NP were validated *in vitro* and *in vivo*. To further optimise biodistribution, one approach, which has been used in both preclinical research and in the clinic, is to pretreat with an unlabelled analogue of the radiopharmaceutical. In the clinic, Bexxar® (^131^I-tositumomab) and Zevalin® (^90^Y-ibritumomab tiuxetan) have been administered following pretreatment with unlabelled tositumomab or rituximab 1, 28. The unlabelled antibody binds to CD20 expressed on normal tissues, which has the effect of reducing the binding of the labelled antibody. This results in decreased accumulation of radioactivity in normal tissues and so reduces the risk of adverse effects. In preclinical studies involving radiolabelled EGF, such strategies have also been exploited to increase tumour uptake while reducing liver uptake 29-31. In this report, we combine a similar strategy with the nanotechnology approach by coadministration of unlabelled targeting ligand instead of unlabelled NP, and study the effect of this coadministration strategy on the tumour and normal tissue uptake of ^111^In-EGF-Au-PEG NP.

## Experimental

### Materials

Recombinant human EGF was obtained from PeproTech (London, UK). Hydrogen tetrachloroaurate(III) hydrate (HAuCl_4_) was purchased from Alfa Aesar (Heysham, Lancashire, UK). ^111^InCl_3_ was purchased from Mallinckrodt (the Netherlands). Cy3-NHS ester was obtained from GE Healthcare (Little Chalfont, Buckinghamshire, UK). Vectashield mounting medium with DAPI was purchased from Vector Laboratories (Peterborough, UK). Thermanox plastic coverslips (13 mm diameter) were purchased from Nalge Nunc International (Rochester, NY, USA). Matrigel was purchased from Corning (Tewksbury, MA, USA). MDA-MB-468 cells were purchased from ATCC. 231-H2N cells were a kind gift from Dr Robert Kerbel. All other chemicals were purchased from Sigma-Aldrich (Dorset, UK).

### Cell culture

MDA-MB-468 and 231-H2N human breast cancer cells were cultured in DMEM with 10% foetal bovine serum (FBS), glutamine (2 mM), and penicillin and streptomycin (100 U/mL). Cells were cultured at 37 °C in 5% CO_2_.

### Synthesis of PEGylated EGF-tagged Au nanoparticles

Diethylenetriaminepentaacetic acid (DTPA)-EGF-Au NP were synthesised using a previously reported method 17. Briefly, 14 nm Au NP were synthesised by citrate reduction of HAuCl_4_. EGF was conjugated with DTPA for subsequent radiolabelling by mixing EGF with a 5-fold excess of cyclic DTPA anhydride in 0.1 M sodium bicarbonate buffer (pH 8.5) and purified by size exclusion chromatography (SEC). To avoid non-specific adsorption of EGF on their surface, Au NP were first dispersed in 0.1% Tween 20, and then incubated with a 160-fold molar excess of DTPA-EGF resulting in generation of Au-S bonds between Au and the disulphide groups of EGF 32. The product, DTPA-EGF-Au, was purified by centrifugation (13,000 rpm, 30 min). PEGylation of DTPA-EGF-Au NP was achieved by adding 10 nmol of poly(ethylene glycol) methyl ether thiol of molecular weight 800, 2000 or 6000 (i.e. HS-PEG800, HS-PEG2000 or HS-PEG6000) to attach to the remaining unoccupied surface of the Au NP after EGF addition. This is known to reduce non-specific EGF adsorption on Au NP 33. The three PEGylated DTPA-EGF-Au NP variants were purified by centrifugation to remove unbound HS-PEG.

### Measurement of hydrodynamic size and zeta potential

The hydrodynamic size (HD) and zeta potential (ZP) of non-PEGylated and PEGylated DTPA-EGF-Au NPs were measured using a Zetasizer Nano (Malvern Instruments). All NP were dispersed in 1 mL of 10 mM NaCl solution (EGF, 0.6 µM; pH ~5.8) for both HD and ZP measurements.

### Radiolabelling of PEGylated EGF-Au nanoparticles with ^111^In

All DTPA-EGF-Au NP, including non-PEGylated NP, were dispersed in 0.1 M sodium citrate (pH 5.5) and incubated with ^111^InCl_3_ for 1 h at room temperature (specific activity: 37.5 MBq/nmol EGF i.e. 6 MBq/µg EGF) to produce ^111^In-EGF-Au and ^111^In-EGF-Au-PEG NP. Quality control was performed using instant thin layer chromatography (ITLC) and phosphorimaging (Cyclone Plus storage phosphor system, Perkin Elmer) with equivalent amounts of InCl_3_ as a control.

### Size-exclusion HPLC analysis

Non-PEGylated and PEGylated NP without removal of unbound PEG-SH were labelled with ^111^In and characterised by size-exclusion HPLC (Waters 2695; Milford, MA, USA) using UV (to detect signal from EGF) and radio detectors. HPLC parameters were as follows: 300 x 7.80 mm Biosep-SEC-S2000 column; mobile phase: pH 7.4 PBS; flow rate: 0.8 mL/min or 0.5 mL/min; 30 min; detecting wavelength 280 nm; room temperature.

### Confocal microscopy

Cy3-labelled EGF was synthesised by reacting Cy3-NHS ester with the primary amine group of EGF in darkness for 2 h, purified using a Sephadex G25 mini-column and attached to Au NP to give non-PEGylated and PEGylated Cy3-EGF-Au NP. MDA-MB-468 and 231-H2N cells were seeded in a Lab-Tek chamber slide (2 x 10^4^ cells/well) and incubated overnight. Cells were then incubated with Cy3-EGF-Au NP (EGF, 40 nM in 200 µL of DMEM containing 10% FBS) for 3 h at 37 °C, washed twice with PBS, fixed using 4% formaldehyde for 10 min at room temperature and then mounted using Vectashield mounting medium with DAPI to stain the cell nuclei. The cells were imaged using a Zeiss 530 microscope (Zeiss, Welwyn Garden City, UK).

### Internalisation assay

MDA-MB-468 and 231-H2N cells were seeded in 24-well plates and incubated overnight (2 x 10^5^ cells/well). Non-PEGylated and PEGylated ^111^In-EGF-Au NP (EGF, 40 nM in 200 µL of growth medium, specific activity: 37.5 MBq/nmol EGF) or equivalent amounts of ^111^InCl_3_ (0.3 MBq in 200 µL of growth medium) were added to wells in triplicate. After incubation for 4 h, the medium containing NP or ^111^InCl_3_ was removed and cells were washed using 0.1 M glycine·HCl (pH 2.5) to remove cell-surface bound radioactivity. Cells were then lysed using 0.1 M NaOH and the internalized radioactivity was counted using an automated gamma counter (Wizard, Perkin Elmer).

### Transmission electron microscopy

MDA-MB-468 cells were seeded on Thermanox plastic coverslips in 24-well plates (2 × 10^5^ cells per well) and incubated overnight. The cells were treated with the DTPA-EGF-Au-PEG6000 NP containing 40 nM EGF for 4 h at 37 °C and washed with 0.1 M PIPES (pH 7.2), followed by fixation using 2.5% glutaraldehyde and 2% paraformaldehyde in 0.1 M PIPES at room temperature for 1 h, then stored at 4 °C until further processing. Samples were washed with 0.1 M PIPES buffer (pH 7.2), post-fixed with 1% osmium tetroxide in 0.1 M PIPES for 1 h at 4 °C, washed with distilled water and incubated with 0.5% uranyl acetate in distilled water overnight at 4 °C. Samples were washed with distilled water and then dehydrated using an ascending alcohol series with 10 min incubations on ice in each of 30, 50, 70, 80, 90 and 95% ethanol, followed by 3 x 20 min incubations in 100% ethanol on ice. Samples were then infiltrated at room temperature with epoxy resin (Agar 100, Agar Scientific) in 100% ethanol as follows: 25% resin for 1 h, 50% resin for 2 h, 75% resin for 1 h and then in 100% resin overnight. After 2 x 3 h incubations in fresh 100% resin, cells were embedded by inverting the coverslip onto a Beem capsule filled with resin and polymerised for 48 h at 60 °C. Blocks were then submerged in liquid nitrogen which allowed the coverslips to be snapped off cleanly, leaving the cell monolayer embedded in the resin. Blocks were sectioned with a Leica UC7 ultramicrotome using a Diatome diamond knife. Sections (90 nm) were collected onto 200 mesh copper grids and post-stained with Reynolds' lead citrate for 5 min 34. Images were acquired using a transmission electron microscope (Tecnai 12, FEI) at 120 kV with a CMOS digital camera (OneView, Gatan).

### SPECT imaging and biodistribution of ^111^In-EGF-Au and ^111^In-EGF-Au-PEG6000 nanoparticles in tumour bearing mice

All animal procedures were carried out in accordance with the UK Animals (Scientific Procedures) Act 1986 and with local ethical committee approval. MDA-MB-468 and 231-H2N xenografts were established in female BALB/c nude mice by subcutaneous injection of 5 x 10^6^ cells (suspended in 1:1 DMEM/matrigel) in the right flank. When xenografts reached a volume of approximately 500 mm^3^, 8 MBq of ^111^In-EGF-Au or ^111^In-EGF-Au-PEG6000 NP with or without unlabelled EGF (15 or 30 µg) was administered intravenously (i.v.) via the tail vein (Table 1). At 24, 48 and 72 h post injection (p.i.), mice were anesthetised using isoflurane and SPECT-CT images were acquired using a nanoSPECT-CT scanner (Bioscan, Washington DC, USA). Region of interest (ROI) quantitative analysis of the SPECT images of the mice bearing MDA-MB-468 xenografts was performed using the InVivoScope software package. After SPECT imaging at 72 h p.i., mice were euthanised and blood, selected tissues, and tumour were removed, weighed and counted for radioactivity in an automated gamma counter. The amount of ^111^In in blood and tissues was expressed as percentage injected dose per gram (%ID/g) of blood or tissue.

## Results and Discussion

### Synthesis of PEGylated ^111^In-EGF-Au NP

NP size has a significant influence on pharmacokinetics, biodistribution and tumour uptake 35. Choi *et al.* studied the blood half-life and biodistribution of PEGylated Au NP with Au-core sizes ranging from 5 nm to 100 nm 36. It was shown that the half-life of PEG (MW 5000)-modified Au NP with core size <20 nm was at least 2-3 times greater than that of larger NP (>20 nm). Similar size-dependent *in vivo* kinetics have also been reported by others 37, 38. A prolonged circulation time would be expected to result in greater accumulation of the NP in the tumour. Increasing the size of PEGylated NP has also been associated with greater accumulation in liver and spleen 35, 36. Zhang *et al.* reported that PEG (MW 5000)-coated Au NP with a core size of 12.1 nm accumulated in tumour to a greater extent than particles with core size of 4.8 nm, 27.3 nm and 46.6 nm 39. Based on these observations, together with a need for optimal radionuclide loading per NP for TRT, we selected Au 14 nm (core size) NP for the current study. We previously reported that this size of Au NP could accommodate 78 copies of ^111^In-EGF per NP 17.

**Figure 1:**
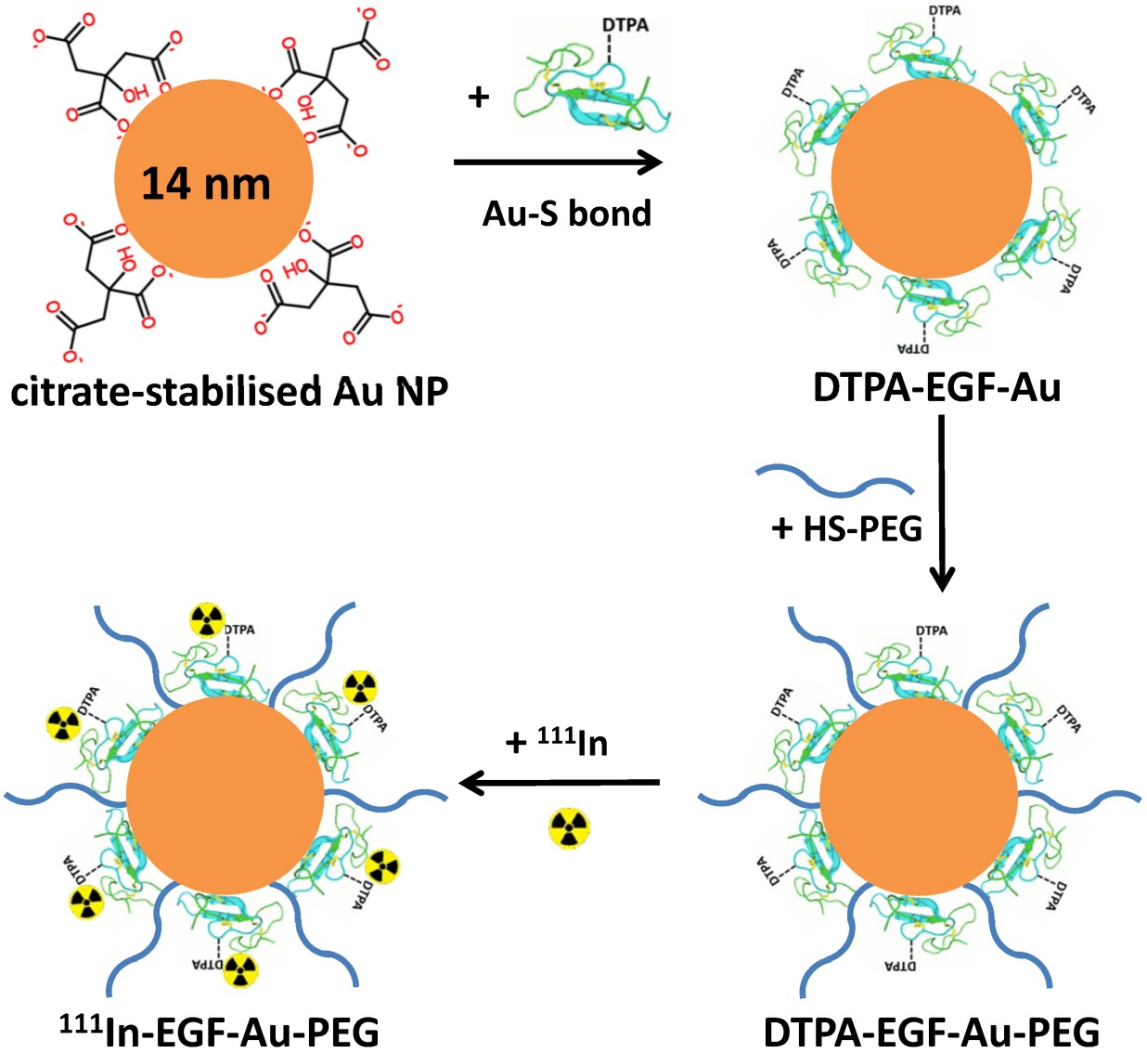
Schematic of the synthesis of ^111^In-EGF-Au-PEG NP. DTPA-EGF was attached to Au NP via Au-S bonds to form DTPA-EGF-Au NP. HS-PEGs were then directly conjugated to the surface of Au NP. Following centrifugation, DTPA-EGF-Au-PEG NP were radiolabelled with ^111^In.

PEGylation of ^111^In-EGF-Au NP was performed prior to ^111^In-labelling by directly attaching HS-PEG to the Au surface of EGF-Au NP via the formation of Au-S bonds (Figure 1). HS-PEGs of different molecular weight, 800, 2000 and 6000, were used to prepare three ^111^In-EGF-Au-PEG NP variants (hereafter referred to as ^111^In-EGF-Au-PEG800, ^111^In-EGF-Au-PEG2000 and ^111^In-EGF-Au-PEG6000). These were compared with non-PEGylated NP in *in vitro* and *in vivo* experiments.

### Characterisation of ^111^In-EGF-Au NP

The HD of DTPA-EGF-Au, DTPA-EGF-Au-PEG800, DTPA-EGF-Au-PEG2000 and DTPA-EGF-Au-PEG6000 was 18.49, 19.38, 24.81 and 32.52 nm, respectively (Table 2 and Figure S3). Although the MW of EGF is 6200 Da, similar to that of the largest PEG (i.e. HS-PEG6000) studied here, all the PEGylated DTPA-EGF-Au NP variants were found to have a greater HD than non-PEGylated NP. This is because the tertiary structure of EGF is such that the contribution of EGF to HD is much less than that of similar-MW PEGs with long flexible polymer chains (HS-PEG2000 and HS-PEG6000), and slightly less than HS-PEG800. As expected, the HD of the NP was shown to increase with increasing molecular weight/chain length of the attached PEG. This observation is consistent with successful PEGylation of the NP.

Non-PEGylated DTPA-EGF-Au NP has a ZP of -24.65 mV. The ZP values for DTPA-EGF-Au-PEG800, DTPA-EGF-Au-PEG2000 and DTPA-EGF-Au-PEG6000 were all smaller than that of DTPA-EGF-Au NP (Table 2). The decrease in ZP after PEGylation results from the charge-shielding effect of PEG 40, 41. The longer the PEG chain the more effective is the shielding it confers. The ZP difference between the non-PEGylated and PEGylated NP is further evidence of successful PEGylation.

The ZP of DTPA-EGF-Au-PEG6000 NP (-9.57 mV) is consistent with other reports of PEG-coated Au NP with sizes ranging from 5 to 100 nm (i.e. -8.44 mV to -12.51 mV) 36. This suggests that the length of the PEG 6000 chain is sufficient to offer an efficient surface charge-shielding effect even in the presence of attached EGF. Surface charge, together with size, shape and surface coating, impacts the *in vivo* fate of NP. It has been shown that highly negatively or positively charged NP can cause undesirable liver uptake while neutral or slightly negative NP (e.g. ~ -10 mV) result in reduced liver uptake and more efficient tumour accumulation 24, 42, 43. This supports the choice of DTPA-EGF-Au-PEG6000, which is slightly negatively charged, for *in vivo* studies.

**Figure 2:**
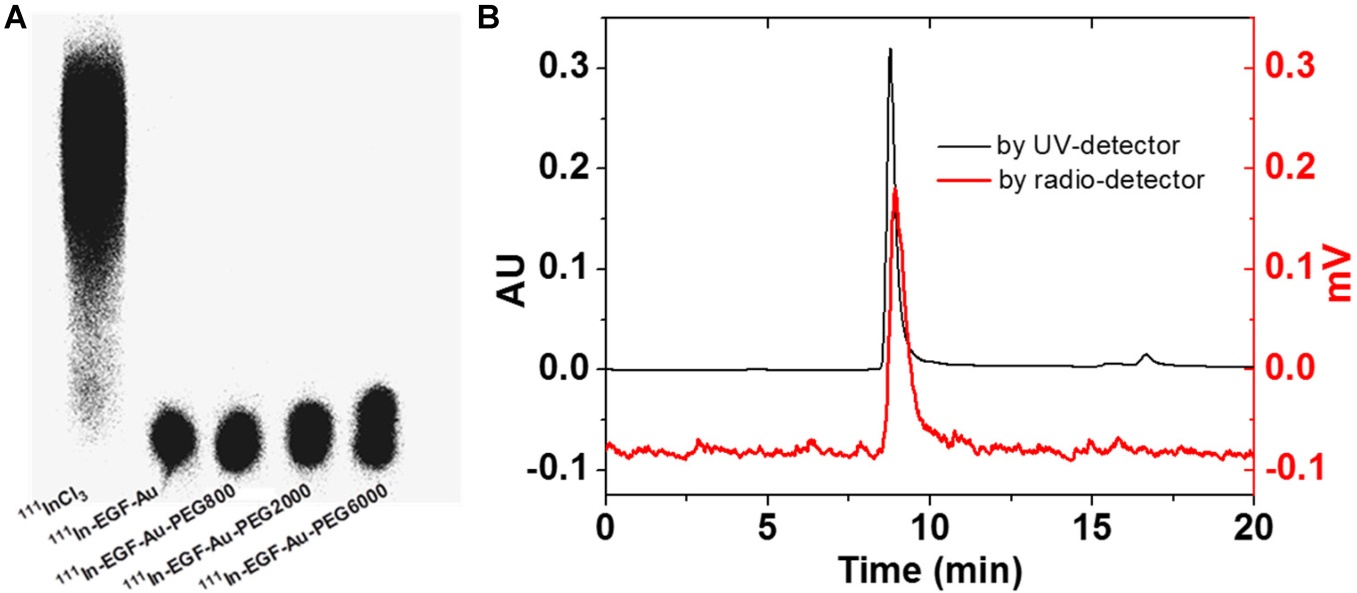
(A) ITLC analysis of ^111^In-EGF-Au NP in comparison to ^111^InCl_3_ visualised by phosphorimaging. (B) HPLC profiles of ^111^In-EGF-Au-PEG6000 using UV- and radio-detection showing that PEGylation does not affect radiolabelling and there is no dissociation of EGF from PEGylated Au NP.

^111^In-labelling of NP was confirmed by ITLC and phosphorimaging (Figure 2A). After incubation with ^111^InCl_3_, the radiolabelling yield of all NP variants was > 90%. Further study of the possible impact of PEGylation on the integrity and radiolabelling of ^111^In-EGF-Au-PEG NP was investigated using size-exclusion HPLC.

HPLC analysis showed that at a flow rate of 0.8 mL/min, the Rt of ^111^In-EGF-Au, ^111^In-EGF-Au-PEG800, ^111^In-EGF-Au-PEG2000 and ^111^In-EGF-Au-PEG6000 was 8.98, 8.97, 8.90 and 8.81 min, respectively (Table 2 and Figure S1). To confirm that the reduced Rt was due to PEGylation leading to greater NP size rather than Rt variation, the flow rate was reduced to 0.5 mL/min. This resulted in Rt values of 11.66, 11.61, 11.48 and 11.39 min for ^111^In-EGF-Au, ^111^In-EGF-Au-PEG800, ^111^In-EGF-Au-PEG2000 and ^111^In-EGF-Au-PEG6000 respectively, demonstrating that increasing NP size after PEGylation results in progressive shortening of Rt (Table 2). The HPLC profile shows that the main peak attributable to ^111^In has the same Rt as that for the NP detected by the UV detector (Figure 2B), confirming that the non-PEGylated and PEGylated NP were successfully radiolabelled and that PEGylation does not have a detrimental effect on radiolabelling efficiency. No additional peak for ^111^In-EGF, the Rt of which was ~14 min (Figure S2), was detected by the UV- or radioactivity detector, showing that EGF remains attached to the NP after PEGylation.

### Cellular studies

**Figure 3:**
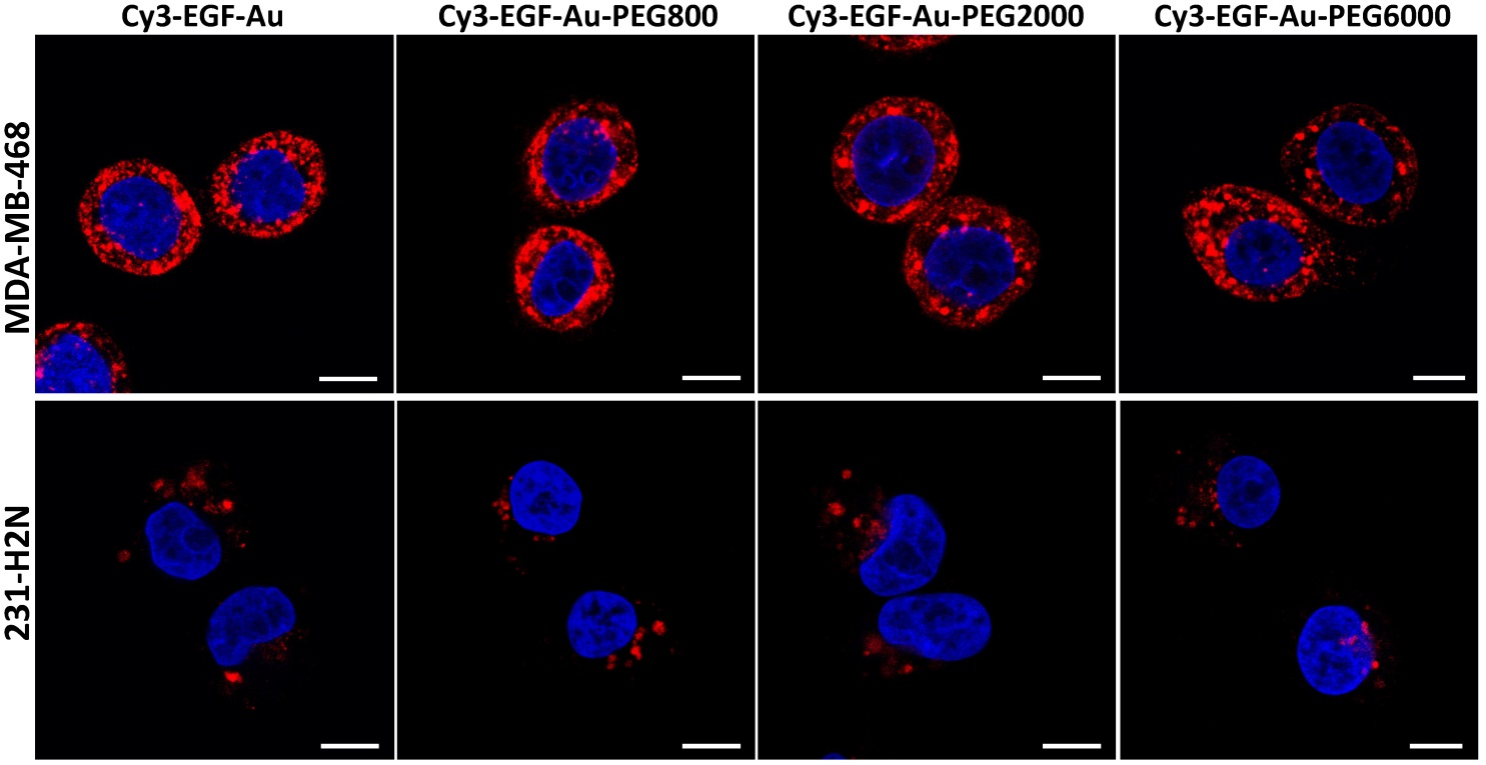
Representative confocal microscopy images of MDA-MB-468 (upper panel) and 231-H2N (lower panel) cells incubated with Cy3-EGF-Au NP or PEGylated Cy3-EGF-Au NP (red) and counterstained with DAPI (blue) (scale bar: 10 µm).

**Figure 4:**
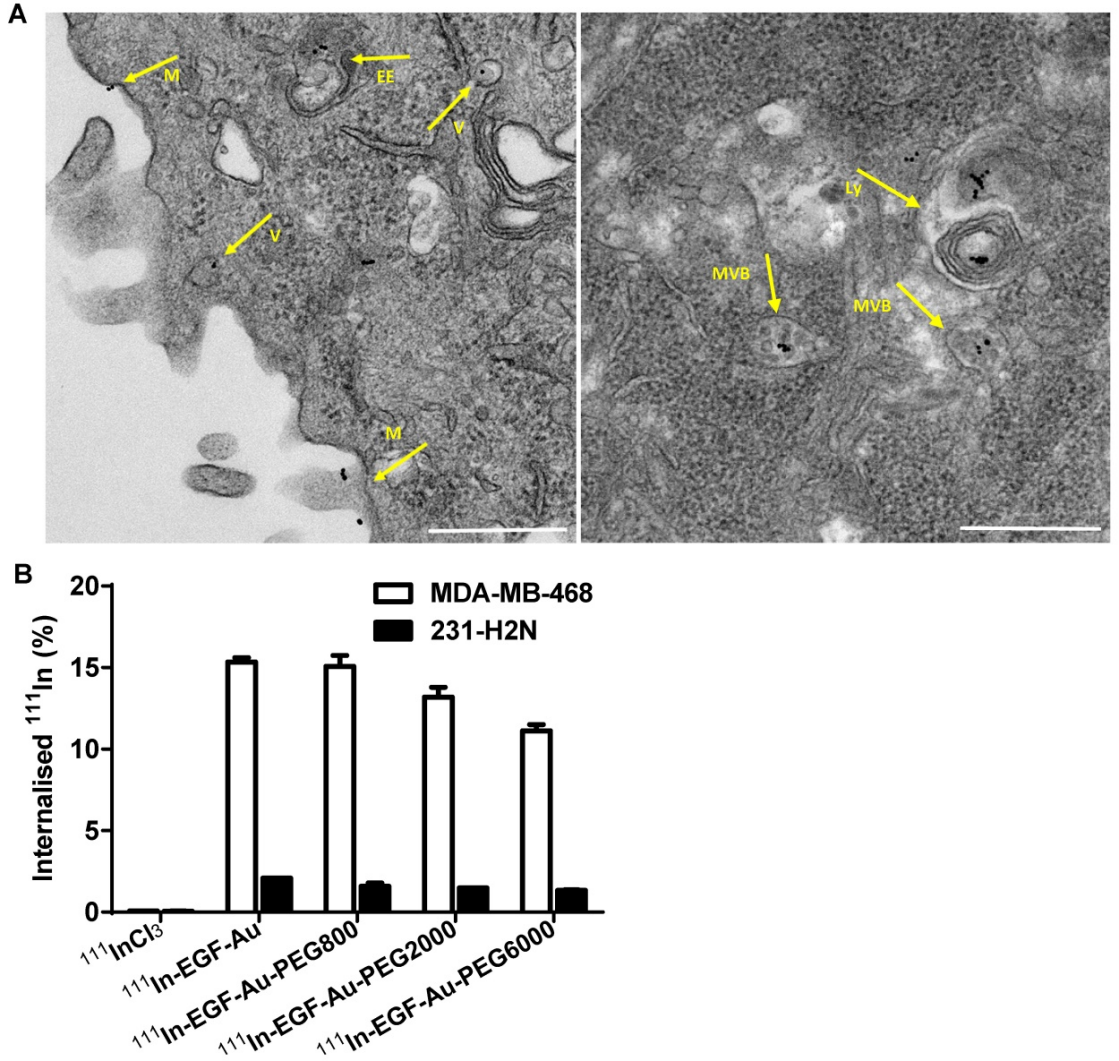
(A) Representative TEM images of MDA-MB-468 cells incubated with DTPA-EGF-Au-PEG6000 for 4 h showing the NP at different stages of the cellular uptake process (M: cell membrane; V: endocytic vesicle; EE: possible early endosome; MVB: multivesicular body/late endosome; Ly: lysosome; Scale bar: 500 nm). (B) Cellular internalisation of ^111^In-EGF-Au and PEGylated ^111^In-EGF-Au constructs (EGF, 40 nM; specific activity: 37.5 MBq/nmol EGF) or equivalent amounts of ^111^InCl_3_ (0.3 MBq in 200 µL DMEM).

Confocal microscopy was used to visualise the cellular uptake of Cy3-labelled NP in MDA-MB-468 and 231-H2N cells (Figure 3, S4 and S5). Incubation with Cy3-EGF-Au and all 3 variants of PEGylated EGF-Au NP resulted in a strong cytoplasmic Cy3 signal in MDA-MB-468 cells. This observation confirms that in the presence of long PEG chains, the NP retain their ability to bind to EGFR with subsequent internalisation of the ligand-receptor complex. There was reduced Cy3 signal in 231-H2N cells, which can be attributed to the approximately 10-fold lower expression of EGFR in this cell line. Transmission electron microscopy (TEM) images of MDA-MB-468 cells incubated with DTPA-EGF-Au-PEG6000 illustrate the different stages of internalisation of single and small clusters of NP (Figure 4A). That the NP cluster in compartments, such as multivesicular bodies/late endosomes, and are not distributed throughout the cytosol, is consistent with the punctate Cy3 fluorescence observed with confocal imaging, particularly in MDA-MB-468 cells. In addition, although it has been shown by us and others that non EGFR-dependent uptake of EGFR-targeting NP may occur 17, 44, we showed previously that non-specific uptake accounts for less than 5.5% of total uptake. Taken together these results suggest that the cellular uptake of the NP occurs mainly through receptor-mediated endocytosis.

To quantify the cellular uptake, internalisation assays were performed using radiolabelled NP (Figure 4B). Following incubation of MDA-MB-468 cells with the ^111^In-EGF-Au constructs, the proportion of total radioactivity that was internalised was 11-15%. In contrast, less than 2% of total radioactivity was internalised by 231-H2N cells. This result reflects the observations made in the confocal experiments described above. Internalisation of NP into MDA-MB-468 cells was reduced as the PEG length of the NP increased (15.3% ^111^In-EGF-Au was internalised, compared to 15.1%, 13.2% and 11.1% of ^111^In-EGF-Au-PEG800, ^111^In-EGF-Au-PEG2000 and ^111^In-EGF-Au-PEG6000, respectively). One possible explanation for this modest reduction in internalisation is that longer PEG chains cause minor steric hindrance that interferes with binding of NP to the EGFR.

Taking the *in vitro* data together, ^111^In-EGF-Au-PEG6000 was selected for *in vivo* experiments. It was considered that the slightly reduced cellular uptake of this construct would be offset by the reduction in opsonisation and, therefore, lower liver uptake, resulting from its effective surface coating and small negative charge. This was expected to result in prolonged circulation time and, therefore, more efficient tumour accumulation compared to the other constructs.

### *In vivo* SPECT imaging and biodistribution studies

**Figure 5:**
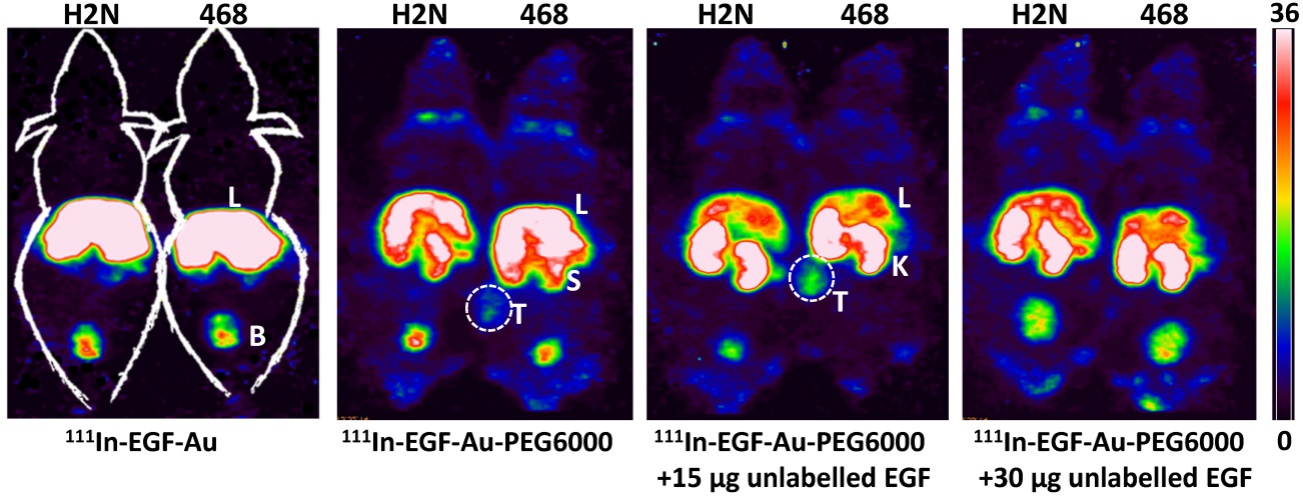
Representative whole-body coronal NanoSPECT images of BALB/c nude mice bearing MDA-MB-468 (468) or 231-H2N (H2N) xenografts at 72 h after i.v. injection of 8 MBq of ^111^In-EGF-Au, ^111^In-EGF-Au-PEG6000 or ^111^In-EGF-Au-PEG6000 plus 15 or 30 µg unlabelled EGF (B: bladder; K: kidney; L: liver; S: spleen; T: tumour).

**Figure 6:**
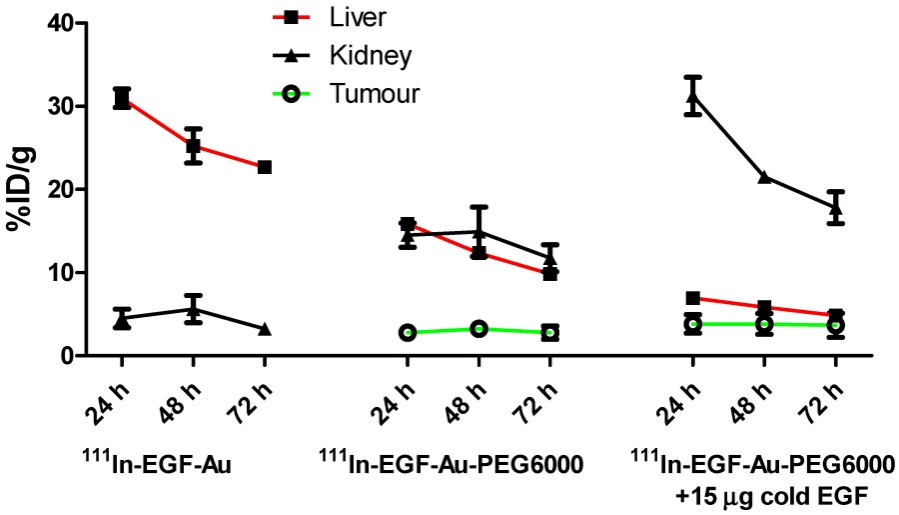
Time-activity curves showing the amount of radioactivity in the liver, kidney and tumour of mice bearing MDA-MB-468 xenografts, derived from quantitative ROI analysis of SPECT images.

**Figure 7:**
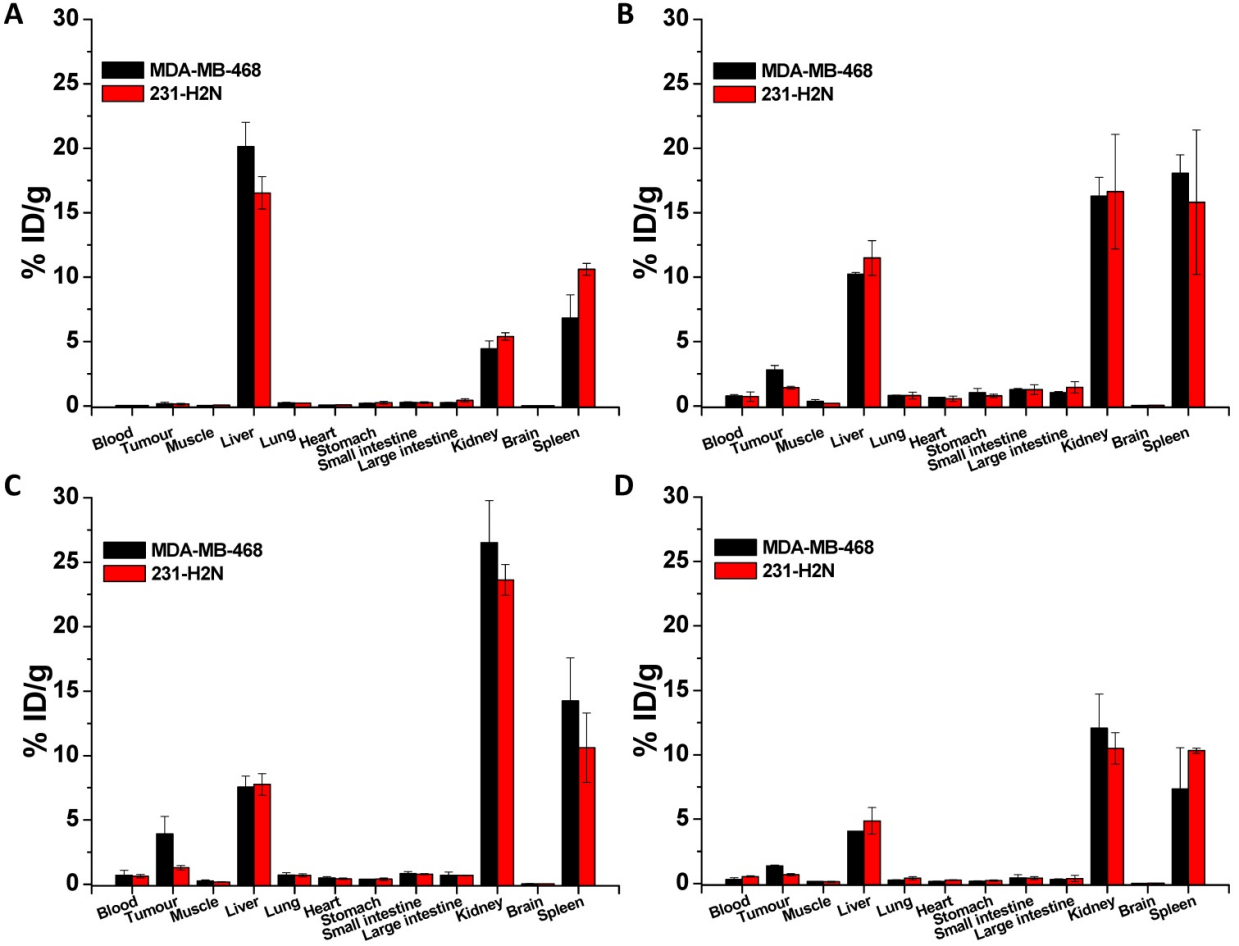
Biodistribution of non-PEGylated ^111^In-EGF-Au (A), ^111^In-EGF-Au-PEG 6000 (B) and ^111^In-EGF-Au-PEG6000 coadministered with 15 and 30 µg unlabelled EGF (C and D) at 72 h p.i. in BALB/c nude mice bearing MDA-MB-468 or 231-H2N xenografts.

BALB/c nude mice bearing MDA-MB-468 or 231-H2N xenografts received intravenous ^111^In-EGF-Au (8 MBq) or ^111^In-EGF-Au-PEG6000 (8 MBq) with or without concurrent unlabelled EGF. Tumour uptake of ^111^In-EGF-Au was not observed in either xenograft model (Figure 5). Volume of interest (VOI) analysis of SPECT images at different time points (Figure 5 and S6) revealed that there was marked hepatic uptake of ^111^In-EGF-Au in MDA-MB-468 xenograft-bearing mice, with 30.97 %ID/g in the liver at 24 h p.i. (Figure 6), and that there was slow clearance of radioactivity from the liver, with 25.23 and 22.69 %ID/g remaining at 48 and 72 h p.i. At 72 p.i. mice were euthanised and tumours and organs removed for measurement of radioactivity. Results (Figure 7A) were consistent with ROI values derived from SPECT images. Following administration of non-PEGylated ^111^In-EGF-Au to mice bearing MDA-MB-468 and H2N-231 xenografts, the liver uptake at 72 h, by direct measurement of radioactivity, was 20.12 and 16.53 %ID/g, respectively. There was also marked renal and splenic uptake (~5 %ID/g and 6-10 %ID/g, respectively) in mice bearing both types of xenograft. The tumour uptake was less than 0.2 %ID/g in both types of xenograft. Without appropriate surface coating, the extensive uptake of the non-PEGylated ^111^In-EGF-Au into the liver and spleen is consistent with the rapid clearance of these NP by the MPS, as reported for other non-coated NP 45, 46.

In contrast, when mice received ^111^In-EGF-Au-PEG6000, there was visible accumulation of radioactivity in MDA-MB-468 xenografts on SPECT scans from 24 h (Figure 5 and S6). Furthermore, liver uptake was 2-fold lower compared to animals that received ^111^In-EGF-Au (Figure 6). Uptake into 231-H2N tumours was less obvious, as expected. The biodistribution data derived from harvested organs were consistent with these findings. After dosing with ^111^In-EGF-Au-PEG6000, the amount of radioactivity that accumulated in MDA-MB-468 xenografts at 72 p.i was 2.81 %ID/g, which was almost 2-fold higher than the amount in 231-H2N xenografts (1.43 %ID/g) (Figure 7B). Furthermore, the liver uptake was approximately 10 %ID/g in mice bearing both types of xenograft, which is 39.5-50.3% lower than the values obtained following administration of non-PEGylated NP. However, the radioactivity uptake in both spleen and kidney was greater than that observed in mice that received non-PEGylated NP.

PEGylation of NP has been shown to confer the ability to evade opsonisation 24, 26, resulting in a lower percentage of NP being taken up by phagocytic cells in the liver. Therefore, the NP remain in circulation for sufficient time to reach the tumour, which accounts for the increased uptake of the PEGylated NP into the MDA-MB-468 xenografts. The increased uptake of NP into the spleen following PEGylation has been observed by others 45. EGFR is reported to be widely expressed (e.g. liver and kidney) 47, 48, and hence the higher kidney uptake could be due to receptor mediated binding of ^111^In-EGF-Au-PEG6000 NP, which would be expected to increase due to the reduction in non-specific uptake by MPS.

Targeting ligands have often been conjugated to the distal end of PEG, to ensure access of the ligand to the targeted cell-surface molecule 49-52. However, since the purpose of PEG is to provide a stealth effect, the presence of the targeting ligand at the distal end of PEG could introduce a new target for opsonins 25, 53. EGF, which is a small peptide, was directly attached to the Au NP surface and 'hidden' by the long-chain PEG (i.e. PEG6000). Interestingly, although PEG had a modest inhibitory effect on EGF-EGFR interaction, PEGylated EGF-Au NP targeted EGFR and were efficiently taken up by MDA-MB-468 cells/xenografts without compromising the PEG stealth effect.

PEGylation can help prevent sequestration of NP by the MPS. However, accumulation in the liver and spleen is still commonly observed for NP with hydrodynamic size of over 8 nm, representing a general challenge for targeted drug delivery nanosystems. This phenomenon may be further compounded by the interaction of the EGF moiety with EGFR in normal tissues, particularly the liver. We investigated whether coadministration of unlabelled targeting ligands (i.e. EGF) could mitigate this effect and found that the *in vivo* fate of ^111^In-EGF-Au-PEG6000 NP, as seen from SPECT scans (Figure 5 and S6) and biodistribution data (Figure 7C and 7D), was indeed altered. Unlabelled EGF (30 µg) inhibited the accumulation of radioactivity in liver, spleen and kidney. However, uptake in MDA-MB-468 xenografts was also decreased from 2.81% to 1.37 %ID/g, confirming that tumour uptake of PEGylated NP is dependent on EGF-EGFR binding. In contrast, when 15 µg unlabelled EGF was co-injected with ^111^In-EGF-Au-PEG6000, the uptake of radioactivity into MDA-MB-468 xenografts was increased by 39.15% to 3.91 %ID/g relative to ^111^In-EGF-Au-PEG6000 alone; while the tumour uptake into 231-H2N xenografts showed little change (1.43 and 1.29 %ID/g in the absence and presence of 15 µg EGF). Coadministration of 15 µg EGF was also associated with a reduction in uptake of radioactivity in the liver and spleen and an increase in uptake into the kidneys compared to administration of ^111^In-EGF-Au-PEG6000 alone. For example, in mice bearing MDA-MB-468 xenografts, liver uptake fell by 25.95% to 7.56 %ID/g following coadministration of 15 µg EGF, whereas kidney uptake increased to 26.51 %ID/g. A possible explanation for these observations is that 15 µg unlabelled EGF is insufficient to block most EGFR but causes partial blockade in liver, leading to a reduction in binding of the radiolabelled NP at this site. This would lead to an increased amount of ^111^In-EGF-Au-PEG in circulation, and so greater accumulation in tumour and kidney 29. Although the optimal amount of coadministered unlabelled EGF has not yet been established, coadministration does appear to offer an effective strategy for radiolabelled targeted nanosystems such as ^111^In-EGF-Au-PEG, leading to enhanced tumour uptake and reduced liver accumulation. The coadministration of unlabelled targeting ligand rather than unlabelled NP (i.e. EGF-Au-PEG6000) reduces unnecessary hepatic accumulation of unlabelled NP.

The results from our study are consistent with those reported by Hu *et al.*, who showed that pretreatment with unlabelled analogues caused an increase in uptake of ^111^In-labelled EGF into EGFR-positive xenografts (from 1.08 to 2.37 %ID/g), with a reduction in liver uptake from 19.58 to 11.19 %ID/g 30. The tumour uptake values in the current study were higher (2.81 and 3.91 %ID/g for ^111^In-EGF-Au-PEG6000 in the absence and presence of unlabelled EGF respectively). This is likely to be a result of the greater payload of radioactivity afforded by the NP delivery system, together with the prolonged circulation time due to the PEGylation. SPECT/CT images show that at 24 h p.i. radioactivity was present in the heart (Figure S6), indicating that PEGylated ^111^In-EGF-Au NP were still in circulation at this time point. These results demonstrate that ^111^In-EGF-Au-PEG6000 NP can deliver radioactivity to tumours more efficiently than ^111^In-labelled EGF. However, coadministration of unlabelled targeting ligand led to higher kidney uptake of the NP, which was also observed in the pretreatment strategy for ^111^In-labelled EGF 29, 30. Further work is needed to explore strategies to reduce NP uptake in kidney.

Accumulation of drugs, including NP-based medicines, in normal tissues may cause undesirable side effects. In some cases additional agents can be used to help reduce such accumulation. Peptide- and antibody-based drugs may show undesirable kidney uptake due to tubular reabsorption after glomerular filtration. This can be inhibited by using positively charged amino acids which neutralise the luminal cell surface of renal tubular cells, resulting in reduced peptide/antibody binding 54, 55. Accumulation in normal tissues is also a major hurdle in the case of nanoparticulate drugs, limiting their progress into clinical use. A similar strategy of coadministration of an agent that alters their biodistribution can be exploited to reduce NP accumulation in healthy tissues. In this report it has been shown that unlabelled EGF can be used to reduce liver uptake of ^111^In-EGF-Au-PEG6000. MPS blocking agents such as gadolinium chloride and empty liposomes have also been reported to show promising results in reducing MPS organ uptake and improving tumour uptake of NP 56-58. Therefore, along with specific tumour-targeting modifications of the NP, the co-delivery of agents that can inhibit uptake in normal tissues or that saturate/inhibit MPS organ uptake, offers a promising direction for the future development of nanomedicines.

## Conclusions

In summary, we have developed radiolabelled PEGylated EGF-tagged Au NP for targeting EGFR-positive cancer. *In vitro* studies confirmed that ^111^In-EGF-Au-PEG6000 binds to and is internalised by EGFR-positive cells. Tumour uptake of ^111^In-EGF-Au-PEG6000 *in vivo* was shown to be enhanced by coadministration of unlabelled targeting ligand, and this was accompanied by a reduction in liver uptake. This offers a potential strategy for developing NP-based TRT for the treatment of EGFR-positive cancer.

## Supplementary Material

Supplementary figures.

## Figures and Tables

**Table 1:** *In vivo* experiment design: Female BALB/c nude mice bearing MDA-MB-468 or 231-H2N xenografts received approximately 8 MBq of ^111^In-EGF-Au, ^111^In-EGF-Au-PEG NP or ^111^In-EGF-Au-PEG NP with 15 or 30 µg unlabelled EGF intravenously, and were then imaged by SPECT/CT at 24, 48 and 72 h.

Group (n=3)	Xenograft	Sample
1	MDA-MB-468	^111^In-EGF-Au
2	MDA-MB-468	^111^In-EGF-Au-PEG6000
3	MDA-MB-468	^111^In-EGF-Au-PEG6000 +15 µg unlabelled EGF
4	MDA-MB-468	^111^In-EGF-Au-PEG6000 +30 µg unlabelled EGF
5	231/H2N	^111^In-EGF-Au
6	231/H2N	^111^In-EGF-Au-PEG6000
7	231/H2N	^111^In-EGF-Au-PEG6000 + 15 µg unlabelled EGF
8	231/H2N	^111^In-EGF-Au-PEG6000 + 30 µg unlabelled EGF

**Table 2:** The HD, ZP and HPLC retention times (Rt) of non-PEGylated and PEGylated NP. Results of HD and ZP are expressed as the mean ± SD of three independent experiments.

Sample	Rt (min) 0.8 mL/min	Rt (min) 0.5 mL/min	HD (nm)	ZP (mV)
DTPA-EGF-Au NP	8.98	11.66	18.49 ± 6.24	-24.65 ± 2.11
DTPA-EGF-Au-PEG800	8.97	11.61	19.38 ± 5.64	-15.37 ± 0.86
DTPA-EGF-Au-PEG2000	8.90	11.48	24.81 ± 7.34	-14.01 ± 0.73
DTPA-EGF-Au-PEG6000	8.81	11.39	32.52 ± 10.56	-9.57 ± 0.59
