# Supplementary material for: Accumulation of 111In-Labelled EGF-Au-PEG Nanoparticles in EGFR-Positive Tumours is Enhanced by Coadministration of Targeting Ligand
Source: Nanotheranostics. 2017 Jun 8;1(3):232–43. doi: 10.7150/ntno.19952 (PMC5646733; doi:10.7150/ntno.19952)
Supplement: Supplementary file 1 — Supplementary figures. [file ntnov01p0232s1.pdf]

Supporting information

Accumulation of ^{111}In-Labelled EGF-Au-PEG Nanoparticles in EGFR-Positive Tumours is Enhanced by Coadministration of Targeting Ligand

Lei Song,[a] Sarah Able,[a] Errin Johnson[b] and Katherine A. Vallis*[a]

[a]CR-UK/MRC Oxford Institute for Radiation Oncology, Department of Oncology, University of Oxford, Oxford, OX3 7DQ, UK

[b]Sir William Dunn School of Pathology, University of Oxford, Oxford, OX1 3RE, UK

*Corresponding author: Prof. Katherine A. Vallis   Katherine.vallis@oncology.ox.ac.uk

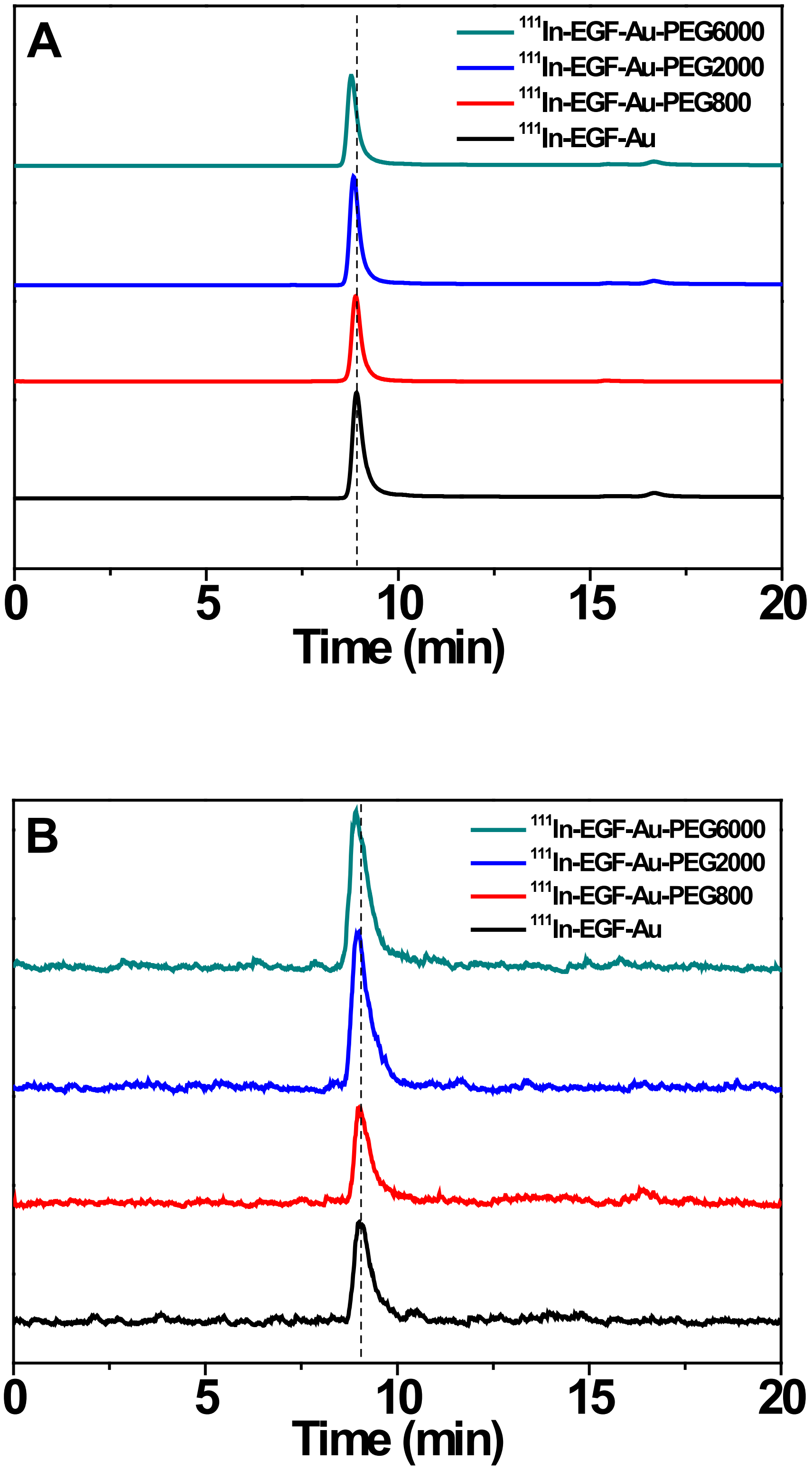

Figure S1. Size exclusion HPLC profiles of ^{111}In-EGF-Au and three ^{111}In-EGF-Au-PEG NP variants (flow rate, 0.8 mL/min) by UV-detection at 280 nm (A) and radio-detection (B), showing that non-PEGylated and PEGylated NPs were successfully radiolabelled and that EGF remains attached to the NPs after PEGylation.

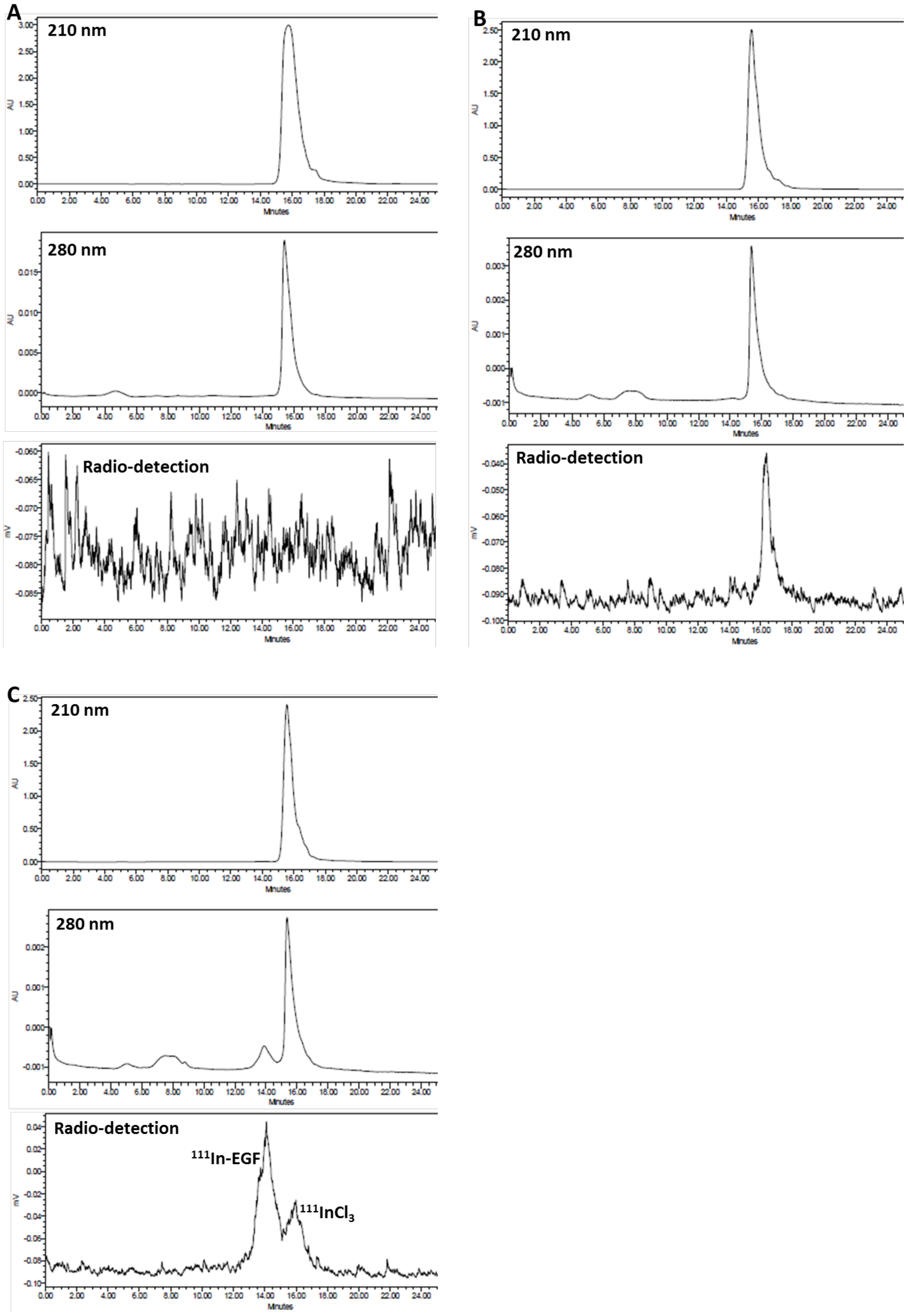

Figure S2. Size exclusion HPLC profiles of 0.1M sodium citrate (A), $^{111}InCl_3$ (B) and ^{111}In-EGF with excess $^{111}InCl_3$ (C) by both UV-detection (210 & 280 nm) and radio-detection.

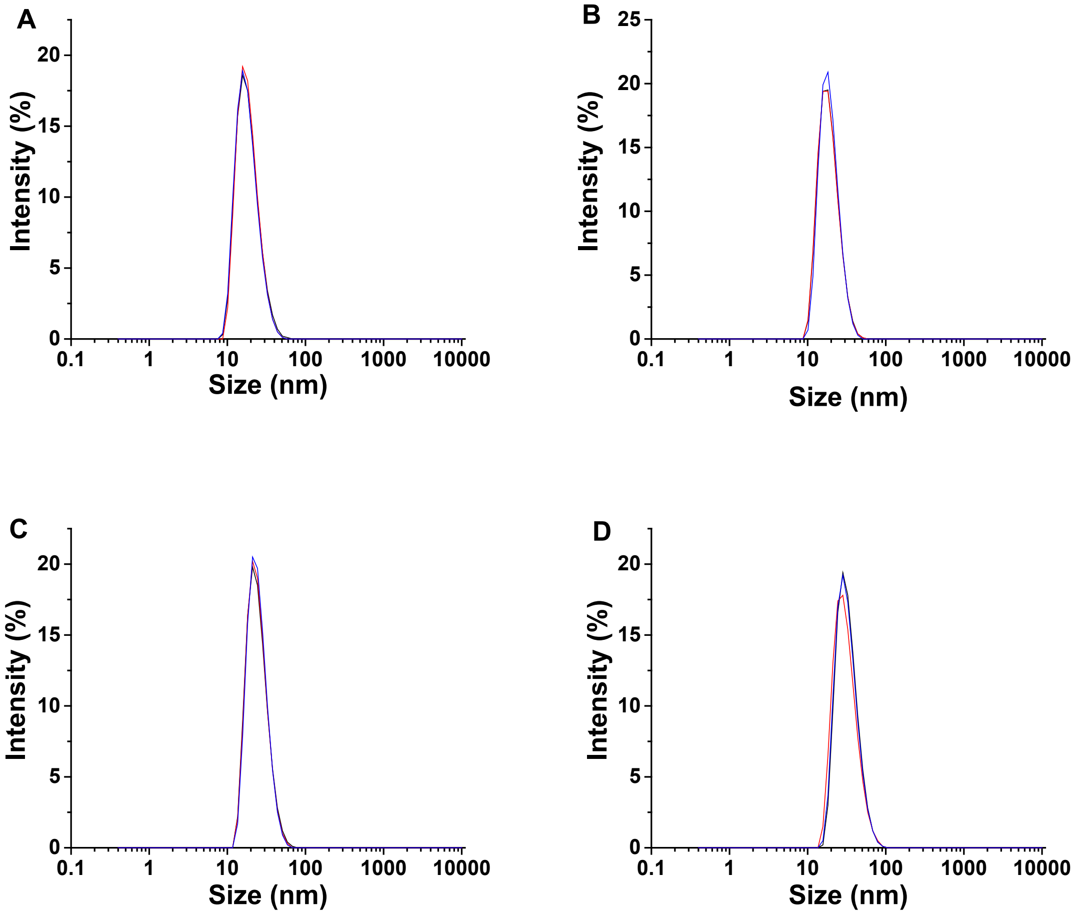

Figure S3. Hydrodynamic size distribution curves of DTPA-EGF-Au (A), DTPA-EGF-Au-PEG800 (B), DTPA-EGF-Au-PEG2000 (C) and DTPA-EGF-Au-PEG6000 (D) NPs from three measurements.

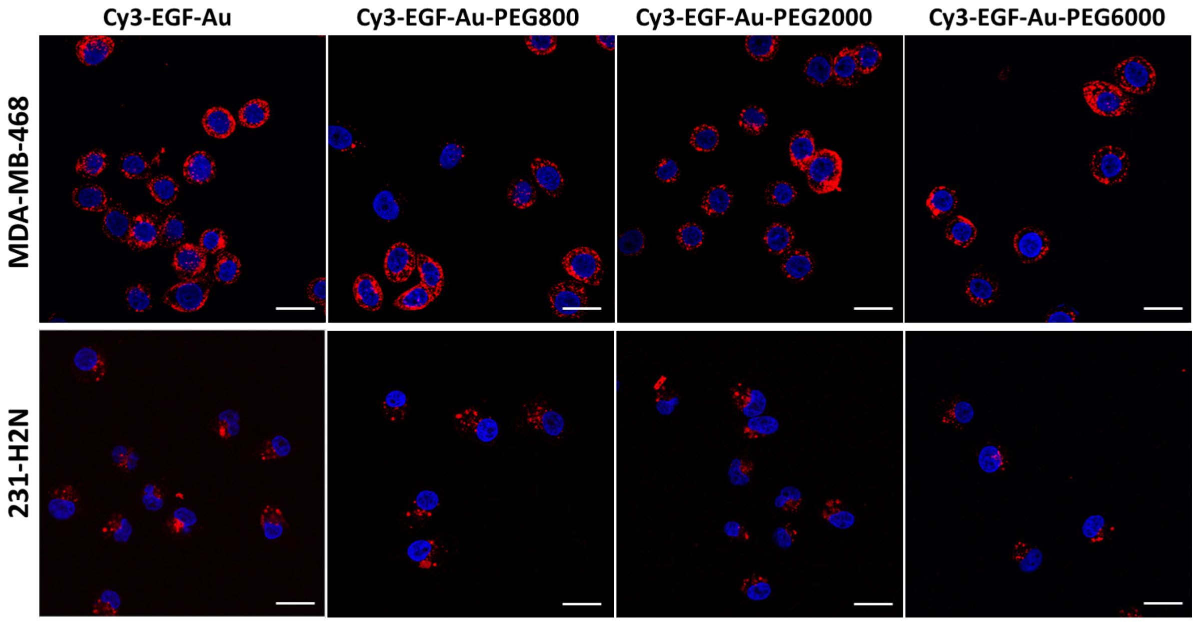

Figure S4. Confocal images of MDA-MB-468 (upper panel) and 231-H2N (lower panel) cells incubated with Cy3-EGF-Au NP or PEGylated Cy3-EGF-Au NP (red) for 3 h at 37°C and counterstained with DAPI (blue) (scale bar: 25 μm).

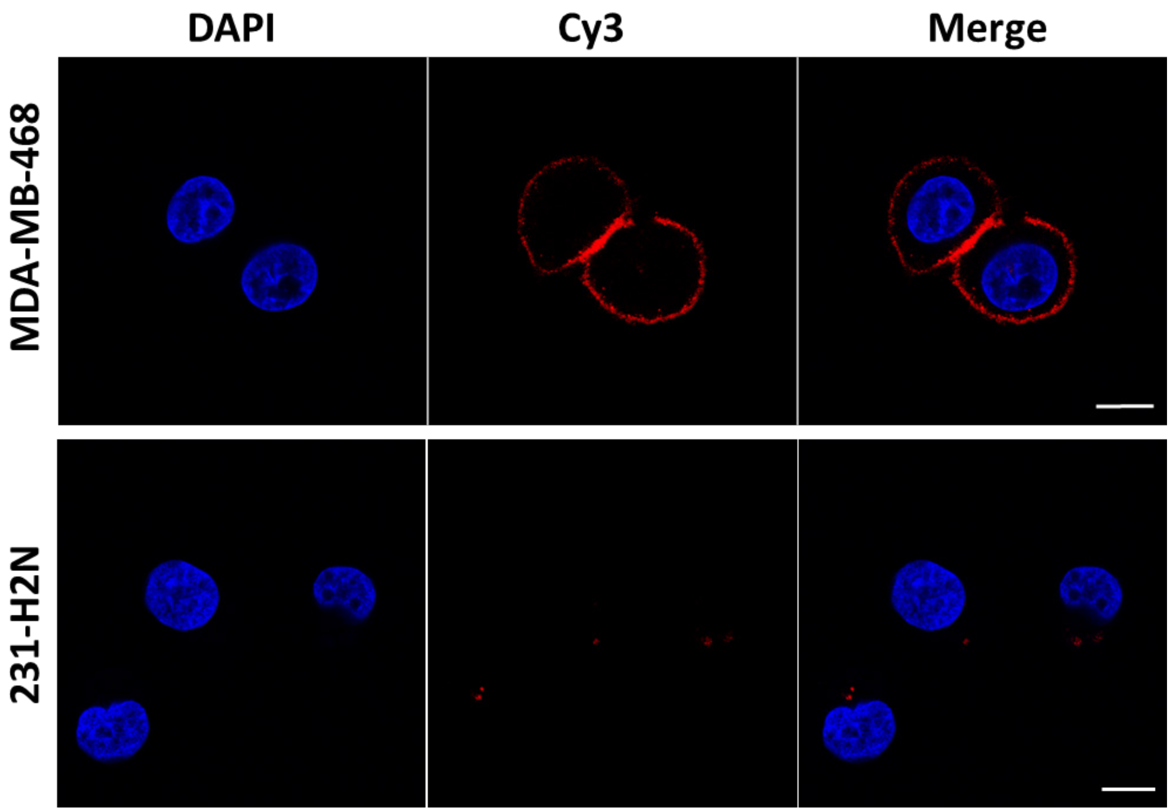

Figure S5. Confocal microscopy images of MDA-MB-468 (upper panel) and 231-H2N (lower panel) cells treated with Cy3-EGF-Au-PEG6000 NPs (EGF, 40 nM) for 3 h at 4°C, showing that Cy3 fluorescence was observed mainly in association with the membrane of MDA-MB-468 cells (scale bar: 10 µm).

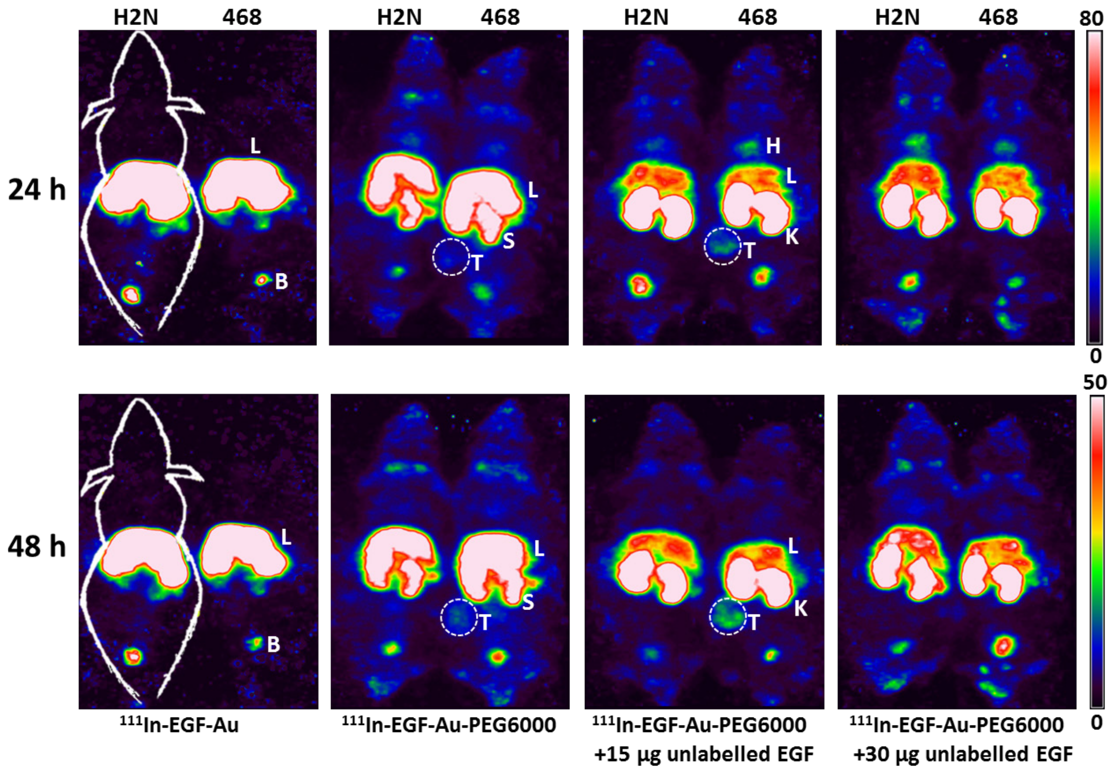

Figure S6. Representative whole-body coronal NanoSPECT images of BALB/c nude mice bearing MDA-MB-468 or 231-H2N xenografts at 24 and 48 h after i.v. injection of 8 MBq of ^{111}In-EGF-Au, ^{111}In-EGF-Au-PEG6000 or ^{111}In-EGF-Au-PEG6000 plus 15 or 30 µg unlabelled EGF (B: bladder; H: heart; K: kidney; L: liver; S: spleen; T: tumour).

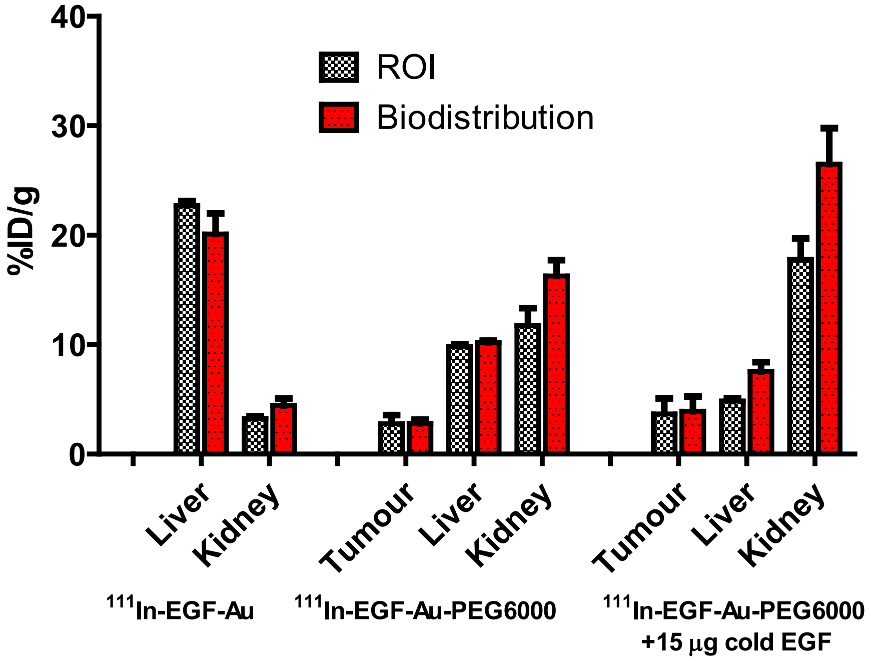

Figure S7. The amount of radioactivity in liver, kidney and tumour at 72 h p.i. in mice bearing MDA-MB-468 xenografts as measured by ROI SPECT image analysis or by gamma counter measurements of harvested tissues.
